# Individual Differences in Adolescent Coping: Comparing a Community Sample and a Low-SES Sample to Understand Coping in Context

**DOI:** 10.1007/s10964-021-01398-z

**Published:** 2021-01-25

**Authors:** Sarah E. D. Perzow, Bethany C. Bray, Martha E. Wadsworth, Jami F. Young, Benjamin L. Hankin

**Affiliations:** 1 University of Denver, Denver, CO, USA; 2 University of Illinois at Chicago, Chicago, IL, USA; 3 The Pennsylvania State University, State College, PA, USA; 4 Children’s Hospital of Philadelphia, Philadelphia, PA, USA; 5 University of Illinois at Urbana-Champaign, Champaign, IL, USA

**Keywords:** Coping, Poverty-related stress, Adolescence, Internalizing psychopathology, Latent profile analysis, Individual differences

## Abstract

Coping that is adaptive in low-stress environments can be ineffective or detrimental in the context of poverty. Identifying coping profiles among adolescents facing varying levels of stress can increase understanding of when and for whom coping may be most adaptive. The present study applied latent profile analysis (LPA) to identify coping profiles in two distinct samples of adolescents: a community sample of youth aged 11–16 years (*N* = 374, *M*_age_ = 13.14, 53% girls), and a low-SES sample of youth aged 12–18 years (*N* = 304, *M*_*age*_ = 14.56, 55% girls). The ten coping subscales of the Responses to Stress Questionnaire were included as indicators in the LPAs (problem solving, emotion regulation, emotion expression, acceptance, positive thinking, cognitive restructuring, distraction, denial, wishful thinking, and avoidance). Five profiles were identified in the community sample: Inactive, Low Engagement, Cognitive, Engaged, and Active Copers. All but the Low Engagement Copers profile were also identified in the low-SES sample, suggesting that adolescents employ similar coping strategies across contexts, but fewer low-SES adolescents engage in lower levels of coping. Profiles differed by gender and symptoms of internalizing psychopathology. Inactive copers in both samples were more likely to be male. Engaged Copers reported the lowest symptom levels whereas Active Copers reported higher symptoms. Cognitive Copers reported higher levels of anxious and depressive symptoms in the low-SES sample only, suggesting that this pattern of coping may be protective only in less stressful contexts. Elucidating within-person coping patterns is a promising avenue for targeting interventions to those most likely to benefit.

## Introduction

Normative stress is an important adolescent experience. Increases in stress exposure and perceived stress are well-established during this period of development (Seiffge-Krenke et al. 2009), and learning to manage stressors is vital to adolescents’ developing cognitive, affective, and social abilities. This necessary process, referred to as “stress-related growth” (Vaughn et al. 2009), contributes to adolescents’ acquisition of broad and flexible repertoires for coping with stress and is an integral part of developing autonomy (Mansfield and Diamond 2015). When levels or types of stress become excessive, however, they can overwhelm an individual’s developing ability to cope. Instead of enhancing development, excessive stress can contribute to the development of psychopathology. Adolescence is a critical period during which incidence rates of internalizing problems such as depression rise dramatically, with evidence that stress exposure plays a role (Grant et al. 2003). Chronic, cumulative, and uncontrollable stressors, such as those associated with living in poverty, contribute to pervasive disparities in mental and physical health outcomes (Wadsworth et al. 2016). The association between stress and internalizing psychopathology that becomes especially apparent in adolescence is complex, however, as substantial individual differences exist regarding who develops psychopathology in highly stressful contexts and who does not. Adolescent coping is implicated in the stress-psychopathology link and may promote adaptation to normative and chronic environmental stress. The present study examines individual differences in patterns or profiles of coping in two distinct samples of youth, a community sample and a low-socioeconomic status (SES) sample, to elucidate similarities and differences in adolescent coping across contexts. This study additionally investigates associations between profile membership and age, gender, and symptoms of internalizing psychopathology to better understand when and for whom coping may be most adaptive.

### Coping: A Malleable Mechanism Linking Stress and Adaptation

**Fig. 1:**
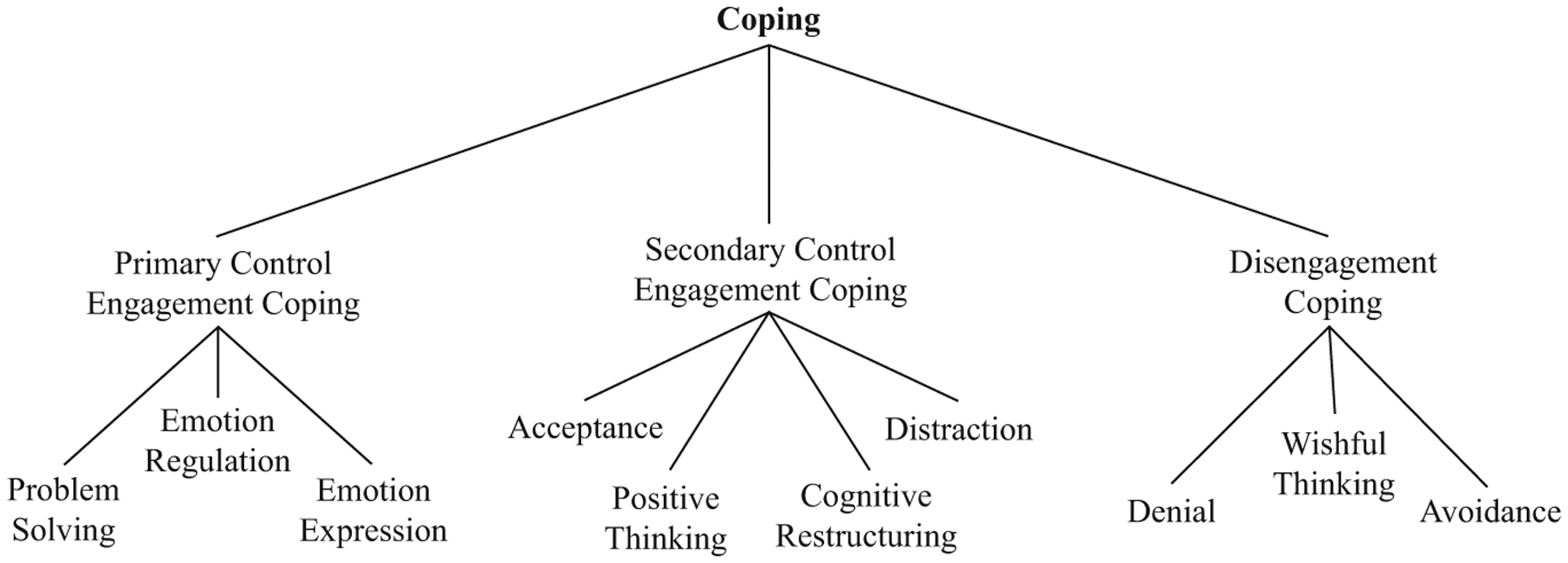
Coping as measured by the RTS

Identifying factors that encourage resilience to adversity may provide critical levers for innovative health promotion and efforts to prevent the development of psychopathology (Card and Barnett 2015). Coping is one such malleable mechanism. Certain coping strategies, for example, can buffer against the consequences of stress exposure, whereas other strategies can exacerbate the negative effects of stress exposure (Compas et al. 2017). The Responses to Stress framework (RTS; Connor-Smith et al. 2000) defines coping as effortful emotional, cognitive, and behavioral responses selected to manage environmental stress that encompass engagement and disengagement strategies (Fig. 1). Primary Control Engagement Coping (PCEC) strategies directly target a stressor or one’s reaction to a stressor and includes social support seeking, Secondary Control Engagement Coping (SCEC) strategies involve adapting or adjusting to stress and includes predominantly cognitive strategies, and Disengagement Coping (DC) strategies are intentional efforts to avoid a stressor or one’s reactions to a stressor. Coping mediates the association between stress and psychosocial adaptation. Wadsworth and colleagues and others have consistently found evidence that PCEC and SCEC strategies are generally associated with greater adaptation whereas DC is linked with poorer psychosocial adjustment (e.g., Evans and Kim 2013; Wadsworth and Compas 2002; Xiao et al. 2010).

### Adolescent Coping with Chronic Stress: Functional Adaptation at a Cost?

The context of poverty creates a chronically stressful environment that constrains normative adolescent psychosocial development and demands greater application of coping than environments characterized by lower levels of stress. Poverty-related stress (PRS; Wadsworth et al. 2011) refers broadly to stressors that are persistently associated with poverty, including economic strain, discrimination, victimization and violence exposure, family transitions or other changes, and family conflict. Chronic PRS exposure may hinder the development of age-appropriate (Zimmer-Gembeck and Skinner 2011) and typically adaptive coping skills (Gonzales et al. 2001), and employing coping strategies that are age-inappropriate (i.e., immature) is associated with increased psychopathology (Auerbach et al. 2010).

Furthermore, there is copious and consistent empirical evidence of socioeconomic disparities in psychosocial adjustment that are evident early in life and persistent across the lifespan (e.g., Evans et al. 2013). Adolescents living in poverty have elevated rates of both internalizing (McLoyd et al. 2009) and externalizing (Schonberg and Shaw 2007) psychopathology, and coping is a recognized mediator of the link between economic strain and adolescent psychosocial adjustment (Wadsworth and Compas 2002). Poverty and other chronic and uncontrollable stressors are not readily managed with strategies like problem-solving that are generally found to be adaptive in less taxing contexts, and consequently PRS may hinder the acquisition of developmentally normative coping repertoires in young adolescents (Gaylord-Harden et al. 2011). Youth facing PRS are likely to rely on more contextually effective and protective avoidance or disengagement strategies (Evans et al. 2005). Although avoidance and disengagement coping is associated with maladaptive outcomes in the long-term for lower-stress samples (Seiffge-Krenke 2000), these responses may be protective in the short-term for adolescents living in a contexts characterized by chronic and uncontrollable stress such as inner-city African American youth (Edlynn et al. 2008) and adolescents coping with family conflict (Santiago and Wadsworth 2009).

Impoverished rural communities represent an understudied and underserved low-SES population in the United States, and stressors associated with rural poverty differ in some ways from urban/suburban PRS. Rates of poverty are higher and employment growth is slower in rural communities as compared to urban areas (United States Department of Agriculture 2019). Rural poverty is associated with lower educational attainment (Byun et al. 2012), geographic isolation (Hudson and Doogan 2019), and health disparities including higher mortality due to physical illness and suicide, higher adolescent birth rates, and higher rates of mental illness (Meit et al. 2014). Further, though Black children are much more likely than White children to live in rural poverty, White rural children who live in poverty may be more likely than Black children to experience psychopathology (Costello et al. 2001). Studying coping among rural, White adolescents is therefore a valuable avenue for future efforts to prevent the development of psychopathology in this specific population.

Similarities and differences in coping repertoires for adolescents with different life experiences can be conceptualized through the lens of functional adaptation, which emphasizes considering the demands of various contexts for understanding the development of competencies such as responding to PRS (Wadsworth 2015). Coping strategies that are protective against environmental threat are more likely to develop in threatening contexts where they are functional, perhaps at the expense of the acquisition of broader and more flexible coping repertoires (Del Giudice et al. 2013). For instance, disengagement coping normatively decreases with development (Amirkhan and Auyeung 2007) but is found to increase with age among youth exposed to chronic PRS (Kim et al. 2016). Disengagement coping strategies may therefore be functionally adaptive in the short-term for avoiding uncontrollable stressors such as poverty, but ultimately may contribute to psychosocial maladaptation over time (Seiffge-Krenke 2004). Holistic measurement of within-person coping patterns can provide information about how disengagement strategies co-occur with engagement strategies, which may offer valuable insight into the temporally adaptive but ultimately maladaptive consequences of different ways that adolescents cope with PRS.

### Associations between Coping, Age, and Gender

Use of different coping strategies may also vary by age and gender. Coping is a developmental process that increases in complexity with age. Children and young adolescents apply more concrete coping strategies such as instrumental problem-solving and distraction, whereas older adolescents and adults use more complex cognitive coping strategies such as self-reliance and planful problem-solving (Zimmer-Gembeck and Skinner 2011). Adolescents and young adults report greater use of SCEC strategies whereas adults report greater use of PCEC strategies, adolescents report greater use of coping strategies overall as compared to young adults (Wadsworth et al. 2004), and adolescents endorse more use of concrete coping strategies such as fundraising, whereas young adults rely more on support-seeking behaviors such as reaching out to friends and family members (Skinner and Zimmer-Gembeck 2007). The consistency of research indicating that adolescent coping repertoires differ from coping in childhood and adulthood highlights the importance of focusing on this period of development to better understand individual differences in coping.

Gender differences in coping are also indicated in adolescence, though research findings are mixed. The gender difference that has the most consistent evidence is the finding that girls report greater use of emotion-based or emotion-focused coping as well as greater coping overall (e.g., Malooly et al. 2017). Some studies suggest that boys report greater cognitive coping strategies as well as more disengagement coping (e.g., avoidance; Eschenbeck et al. 2007), whereas other studies indicate that boys do not report higher use than girls on any coping scales (Connor-Smith et al. 2000). Girls have also been found to endorse greater engagement in coping overall (Wadsworth et al. 2004) and in use of social coping strategies such as fundraising, talking to others, and attending church services specifically (Persike and Seiffge-Krenke 2012). Greater empirical attention is needed to better understand whether gender differences in coping are consistent across contexts.

### Person-Centered Methods for Studying Coping

Coping has predominantly been conceptualized as a continuous latent construct, with individuals engaging in a variety of related behavioral strategies at different levels. Importantly, however, coping is a multifaceted process in which many strategies across categories or domains of coping interact rather than occurring in isolation (Skinner et al. 2003). Adolescents who use a combination of engagement and disengagement coping strategies likely have different psychological outcomes than adolescents who rely exclusively on disengagement strategies. Person-centered approaches such as latent class analysis, latent profile analysis (LPA), and latent transition analysis that model interactions among variables within individuals can be used alongside traditional variable-centered methods to identify common combinations of coping strategies that adolescents employ. This approach to studying coping patterns can provide valuable insight into this process and offer guidance for promoting adaptation in the face of stress during adolescence. Uncovering these within-person patterns will aid in developing more cost-effective, feasible, and effective prevention and intervention programs by helping to target interventions or intervention components to individuals most likely to benefit.

Few studies have investigated individual differences in how adolescents cope, but the ones that do contribute valuable information for understanding adolescent adaptation. Some combinations of strategies appear to be more effective than others, and these patterns cannot be uncovered using traditional variable-centered approaches. For instance, Herres (2015) identified four coping profiles in a community sample of adolescents. These profiles were labeled as Disengaged Copers (16.1%), Independent Copers (39.5%), Social Support Seekers (31.6%), and Active Copers (12.8%). Disengaged Copers endorsed greater use of avoidance coping strategies such as humor and mental disengagement as compared to approach-oriented coping strategies such as active coping, planning and seeking social support. Independent Copers reported less reliance on interpersonal strategies such as venting and social support seeking and greater use of more independent strategies such as humor and planning. Social Support Seekers reported higher use of both emotional and instrumental support seeking. Active Copers reported greater use of all coping strategies and reported especially high reliance on approach coping strategies including active coping, planning, and social support seeking. Though Independent Copers engaged in less social support seeking, a coping behavior that traditional variable-centered methods conceptualize as adaptive, they also reported the fewest depressive symptoms. Two other profiles identified in this study that likely would be hypothesized to have lower levels of psychopathology based on findings from variable-centered studies—Social Support Seekers and Active Copers—in fact reported the highest levels of anxiety. A small body of literature applying person-centered approaches to the study of coping have led to similar conclusions (Aldridge and Roesch 2008; McDermott et al. 2019; Okafor et al. 2016), indicating that traditional variable-centered approaches to classifying coping may be missing vital associations between combinations of coping strategies and psychosocial outcomes.

Even fewer empirical examinations of adolescent-specific patterns of responses to PRS have been published to date. In one notable example, Aldridge and Roesch (2008) took a person-centered approach to measuring dispositional coping styles in low-income adolescents of color. The authors identified three profiles: Low Generic Copers (44.4%, Active Copers (48.3%), and Avoidant Copers (7.3%). Low Generic Copers reported lower than average use of all coping strategies measured in this study. Active Copers reported reliance on more active strategies (active coping, planning, instrumental social support seeking) and less use of avoidant strategies. Avoidant Copers reported reliance on passive or avoidant strategies (denial, behavioral disengagement, substance use) and endorsed lower use of active coping strategies. Active Copers were slightly but significantly older than the other two profiles, and gender was not significantly associated with profile membership. Avoidant Copers endorsed significantly more depressive symptoms than the other two profiles. Adolescents in the Active Copers profile reported fewer depressive symptoms and greater stress-related growth than the other two profiles, and adolescents in the Low Generic Copers profile reported fewer symptoms of depression but also experienced less stress-related growth than the Active Copers. The Low Generic Copers profile is a group that had previously been unidentified in the adolescent coping literature. The authors posited that the Low Generic Copers adolescents may still be acquiring coping repertoires. In other words, these adolescents may have less mature coping skills than other adolescents in the study. Low Generic Copers were younger than the other study participants and the authors’ speculation of immaturity in their coping repertoire may be accurate. Alternatively, these adolescents may simply perceive less stress in their lives and thus face fewer opportunities to cope. There may also be gender differences in coping reports that could contribute to the identification of this profile. Research with low-SES parents indicates that these demographic differences are likely—Perzow et al. (2018) identified a Low Responders profile who reported low levels of all stress responses, and members of this profile were more likely to be men and to report lower levels of perceived stress.

Person-centered methods such as LPA can be used to identify homogeneous subgroups of adolescents within a heterogeneous sample based on multiple shared characteristics (Herman et al. 2007). As the previously reviewed studies indicate, findings from studies that investigate individual differences in coping patterns or profiles often uncover surprising associations that may not be expected based on findings from variable-centered studies. This methodology can expand current understanding of how, when, and for whom certain coping strategies are adaptive (Lanza and Rhoades 2013), and can inform patient-centered treatment and prevention efforts by matching groups to the most appropriate treatment approaches (Cooper and Lanza 2014).

### Current Study

The present study used LPA to compare coping profiles in a nationally representative community sample (the Genes, Environment, Mood (GEM) study) to coping profiles in a rural, low-SES sample of predominantly White adolescents (Adolescents Coping with Poverty project). As with many applications of LPA today, an exploratory perspective is used. However, this approach is not atheoretical, nor is it completely data-driven: informal hypotheses about the general nature of several expected profiles are provided for context and to situate the current study within existing literature.

The first aim of the present study was to identify and describe coping profiles in these two distinct samples of adolescents (the community sample and the low-SES sample). There is a dearth of studies elucidating individual differences in patterns of coping in youth; the few existing studies have generally uncovered 3–4 profiles that were distinguished by different combinations of approach and avoidance coping strategies. All previous studies have identified one profile that endorsed low use of all coping strategies and one profile characterized by higher use of active or approach-oriented coping strategies. Given clear similarities in patterns or profiles identified in previous studies, it was hypothesized that the same profiles would be identified in both samples. Consistent with previous research, it was hypothesized that one profile characterized by low levels of all coping strategies and one profile characterized by high levels of engagement strategies and low levels of disengagement strategies would be identified in both study samples. Importantly, chronic and uncontrollable stress such as economic strain and family conflict are associated with greater disengagement coping and less reliance on engagement coping. Though the low-SES sample is expected to face higher levels of chronic stress associated with poverty, it is likely that at least some of the adolescents from the community sample also face some chronic and/or uncontrollable stress. Therefore, it was hypothesized that a third profile reporting high levels of disengagement coping strategies and low levels of engagement coping strategies would be identified in both samples.

The second aim of the present study was to compare the coping profiles identified in these distinct samples of adolescents to determine similarities and differences in how coping may develop across contexts. As elaborated in Aim 1, it was hypothesized that the same profiles would be identified in both samples. However, the context of poverty creates a chronically stressful environment that constrains normative adolescent psychosocial development and demands distinctive coping efforts. It was therefore hypothesized that profile prevalences would be different in the low-SES sample as compared to the same profiles identified in the community sample. Profile(s) reporting lower levels of coping overall were expected to be smaller in the low-SES sample, whereas profile(s) reporting high levels of all coping strategies or high levels of disengagement coping strategies were expected to be larger in the low-SES sample.

The third and final aim of the present study was to examine descriptive differences by age, gender, and internalizing psychopathology. As coping repertoires normatively become increasingly complex with age, profile membership was hypothesized to differ by participant age. Further, because previous studies have indicated gender differences in coping, profiles were hypothesized to differ by gender. Finally, because coping and psychopathology are consistently and clearly linked in the literature, it was hypothesized that profiles would differ on measures of psychopathology.

## Methods

### Participants and Procedures

This study involved secondary data analysis using data from the GEM study (the community sample; see Hankin et al. 2015) and the Adolescents Coping with Poverty project (the low-SES sample; see Wadsworth and Compas 2002). All procedures were approved by the institutional review boards at each study site. Informed consent was obtained from parents/legal guardians.

The GEM Study was a longitudinal multiwave study conducted at the University of Denver and Rutgers University (see Hankin et al. 2015). Data were collected between September 2008 and June 2014. Participants were initially recruited in the third, sixth, and ninth grades and were followed prospectively for three years. Families living near the University of Denver (Denver metro area) and Rutgers University (central new Jersey area) were sent brief informational letters and interested families contacted respective research teams to participate. Youth (*N* = 665, 55% girls) ranging in age from 7 to 16 years at baseline (*M* = 11.6, *SD* = 2.4) and one self-selected caregiver (85% mothers) were recruited via letters sent home from school. Interested families contacted the laboratory and were screened for eligibility. The GEM Study sample was comparable to the community and school districts in the areas of recruitment. Specifically, the median annual income of families in the GEM study sample was $86,500 and 18% reported receiving free/reduced lunch. The majority of participants parents were married (77%), 15% were divorced or separated, and 7% were single. The GEM study sample was generally representative of the racial-ethnic demographic of the United States during the time of data collection (62.2% White, 11.3% African American, 7.5% Hispanic, 9.6% Asian/Pacific Islander, 9.3% other/mixed race or ethnicity). Eligible families were fluent in English, and youth were not diagnosed with an autism spectrum or psychotic disorder and had an IQ > 70.

The present study included all available data from GEM participants recruited in sixth and ninth grades (*N* = 484) because of the questionable reliability of youth self-report prior to age 11 (Ebesutani et al. 2011), and used cross-sectional questionnaire data collected at the baseline assessment (*M*_age_ = 13.14, *SD*_age_ = 1.62; 53% girls). Baseline data collection took place from September 2008 to June 2011. Only participants missing all data on the coping questionnaire were excluded from analyses (analysis sample *N* = 374). Little’s (1986) test was non-significant (*χ*^2^[81] = 78.05, *p* = 0.57) for the coping questionnaire, suggesting that data were missing completely at random. Additionally, there were no differences between participants missing all coping data and participants with some coping data on measures of age, ethnicity, family income, or measures of psychopathology (*p*’s > 0.05); however, participants missing all coping data were more likely to be male and African American/Black (*p*’s < 0.05). This sample is referred to as the community sample.

The Adolescents Coping with Poverty project (Wadsworth and Compas 2002) was a cross-sectional study that investigated stress responses as a moderator and mediator of adolescent psychological adjustment to PRS. Data were collected between September 1996 and June 1998. Youth (*N* = 364, 55% girls) ranging in age from 12 to 18 years (*M*_*age*_ = 14.56, *SD*_*age*_ = 1.65) were recruited from a middle school and high school in rural northeastern New England. At the time of recruitment, the county where participants were living had the second highest unemployment rate in the state. Twenty-nine percent of the sample was enrolled in the district’s free/reduced lunch program (which is likely an underestimate of the degree of poverty in the sample, as many families in rural areas do not enroll in these programs even though they qualify for them). The majority of participants (69.5%) were living in two-parent households, 20.5% were living in one-parent households, and 10% reported not living with their parents. Family SES was indexed using the Hollingshead (1975) 9-point parental employment scale (1 = lowest level of employment); the mean SES of the sample was 3.7, suggesting that parents of participating youth worked as laborers or farmers, for example, if they were employed. The sample was representative of the local racial-ethnic demographic (97% White). Only participants missing all data on the coping questionnaire were excluded from analyses (analysis sample *N* = 304). Little’s (1986) test was non-significant (*χ*^2^[27] = 20.67, *p* = 0.80) for the coping questionnaire, suggesting that data were missing completely at random. There were no differences between participants missing all coping data and participants with some coping data on measures of age, gender, parent education level, parent occupation, or measures of psychopathology (*p*’s > 0.05). This sample is referred to as the low-SES sample.

### Measures

#### Demographics

For the community sample, caregivers reported demographic information including their child’s age, gender, race, and ethnicity, as well as family composition and socioeconomic status. Adolescents in the low-SES sample self-reported on personal demographic information including their age, gender, race, ethnicity, and parents’ education and employment.

#### Coping

Both studies measured coping using the Responses to Stress Questionnaire (RSQ; Connor-Smith et al. 2000), a self-report questionnaire that consists of 57 items rated on a scale from 1 (*not at all true*) to 4 (*very true*). Items load onto three voluntary coping factors (PCEC, SCEC, and DC) and two involuntary stress response factors (involuntary engagement and involuntary disengagement). Voluntary stress responses (i.e., coping) are effortful, conscious reactions to stress. In contrast, involuntary stress responses are automatic, unplanned responses to stress that are not under an individual’s control (e.g., physiologic arousal, intrusive thoughts, emotional numbing, inaction). The five-factor structure of the RSQ has been established in numerous and diverse samples (e.g., Benson et al. 2011; Xiao et al. 2010), and the subscales have shown adequate internal consistency (*α*’s = 0.67–0.92; Connor-Smith et al. 2000).

The present study included the ten voluntary coping subscales that index PCEC, SCEC, and DC as indicators of coping profiles; this study did not investigate involuntary stress responses. A total of 37 items assess seven engagement coping strategies (PCEC: problem solving [e.g., “I try to think of different ways to change the problem or fix the situation”], emotion regulation, [e.g., “I keep my feelings under control when I have to, then let them out when they won’t make things worse”], emotion expression [e.g., “I let someone or something know how I feel”]; SCEC: acceptance [e.g., “I realize that I just have to live with things the way they are”], positive thinking [e.g., “I tell myself that everything will be alright”], cognitive restructuring [e.g., “I think about the things that I’m learning from the situation, or something good that will come from it”], and distraction [e.g., “I think about happy things to take my mind off the problem or how I’m feeling”]) and three disengagement strategies (DC: denial [e.g., “When I’m around other people, I act like the problems never happened”], wishful thinking [e.g., “I deal with the problem by wishing it would just go away, that everything would work itself out”], and avoidance [e.g., “I try to stay away from people and things that make me feel upset or remind me of the problem”]). The community sample completed a shortened version of the RSQ including only the 37 items assessing voluntary stress responses (i.e., coping); internal consistency of the RSQ for the community sample was excellent (*α* = 0.92). The low-SES sample completed the full RSQ; internal consistency was good (αs = 0.80–0.85). Scale scores for the ten coping subscales for both samples were used in analyses (Fig. 1).

#### Psychopathology

Psychopathology was measured differently in these two distinct studies. Measures of internalizing psychopathology that were available for each sample are included in order to investigate whether internalizing symptoms were associated with profile membership.

##### *Depressive symptoms* (community sample)

Youth in the GEM Study reported on symptoms of depression using the Children’s Depression Inventory (CDI; Kovacs 1985), a self-report questionnaire consisting of 27 items rated on a 0–2 scale; higher scores indicate greater symptom endorsement. The CDI is a frequently used measure that has demonstrated internal consistency, test-retest reliability, and validity in samples of children and adolescents. Internal consistency for the community sample was good (*α* = 0.85). The sum, representing number and severity of symptoms endorsed, was used in analyses.

##### *Anxiety symptoms* (community sample)

Youth in the GEM Study reported on symptoms of anxiety using the Multidimensional Anxiety Scale for Children (MASC; March et al. 1997), a self-report questionnaire consisting of 39 items rated on a scale from 0 (*never true about me*) to 3 (*often true about me*). Items load onto four subscales: physical symptoms, social anxiety, separation/panic, and harm avoidance. The MASC is a frequently used measure that has established internal consistency and test-retest reliability validity in samples of children and adolescents. Internal consistency for the MASC composite score in the community sample was good (*α* = 0.88). The sum for all subscales, representing total number and severity of symptoms endorsed, was used in analyses.

##### *Internalizing symptoms* (low-SES sample)

Youth in the Adolescents Coping with Poverty project reported on emotional and behavioral problems using the Youth Self Report (YSR; Achenbach, 1991), a self-report measure consisting of 112 items rated on a scale from 0 (*never true*) to 2 (*very often true*). The YSR is a frequently used measure with established reliability and validity. Internal consistency for the low-SES sample was good (*αs* = 0.86–0.90). The *T*-score for the anxious/depressed symptoms scale was used in analyses.

### Analysis Plan

The data analytic plan and hypotheses for the present study were preregistered with Open Science Framework (https://osf.io/47v2n/;https://osf.io/7sd3e/). Data analysis proceeded in two phases. First, latent profiles of coping were identified and described in the community sample and the low-SES sample using LPA. Second, members of the identified latent profiles in each sample were characterized by examining differences in their composition based on age, gender, and internalizing psychopathology.

In LPA, two sets of parameters are of primary interest: (1) latent profile membership probabilities (i.e., pre-valences) that describe the distribution of profiles, and (2) item-response means (and variances) that provide profile-specific means (and variances) of the coping strategies endorsed. Item-response means are used to interpret and label the profiles. Model selection was guided by the Akaike information criterion (AIC; Akaike 1974), Bayesian information criterion (BIC; Schwarz 1978), sample size adjusted BIC (a-BIC; Sclove 1987), Vuong-Lo-Mendell-Rubin Likelihood Ratio Test (VLMR; Vuong 1989), Bootstrap Likelihood Ratio Test (BLRT; McLachlan 1987), and entropy (Celeux and Soromenho 1996), as well as model stability and interpretability. Theoretical and clinical interpretation was emphasized in model selection because, as is common in LPA (see e.g., Bray et al. 2014; Fosco and Bray 2016), model fit indices were expected to continue to decrease as additional profiles were added. Wald tests were used to identify significant differences between each item response mean and the overall sample means (Asparouhov 2007). Model estimation was conducted using Mplus versions 7.4–8.0 (Muthén and Muthén 1989–2018); model identification for all LPA models was checked using 1000 initial stage starts and 100 final stage starts.

Associations between coping profile membership and descriptive measures including age, gender, and psychopathology were examined in both samples. These associations are expressed as pairwise differences in profile-specific means for age and psychopathology and pairwise differences in proportions of boys and girls in each profile. These comparisons were conducted using the Bolck, Croon, and Hagenaars (BCH) approach, which assigns participants to profiles based on their modal posterior probabilities and then adjusts for classification error in these assignments when estimating the profile-specific distributions (Bolck et al. 2004; Bakk and Vermunt 2016).

## Results

Models with 1–8 profiles were considered for both samples. Table 1 presents model fit and selection information. Models with greater than 8 profiles were not considered due to difficulty with model identification. In both samples, all models assumed multivariate normality of indicators conditional on profile and equal indicator variances across profiles; without the equal variances assumption many sets of starting values were unable to converge, making model identification difficult to ensure, and non-positive definite first-order derivative product matrices prevented some standard errors from being estimated.

### Community Sample

Model fit information and selection criteria for the latent profile models of coping suggested five profiles appeared superior to models with fewer or greater profiles. This supports the hypothesis that at least three coping profiles would be identified. The 5-profile model showed greater profile separation compared to the 4-profile model, and the additional profile in the larger model improved interpretation. In contrast, when moving from the 5-profile model to the 6-profile model, one of the profiles was split into two smaller profiles with similar interpretations. A similar split happened when moving from the 6-profile model to the 7-profile model. Thus, the 5-profile model was selected as optimal for interpretation and further analysis.

**Fig. 2:**
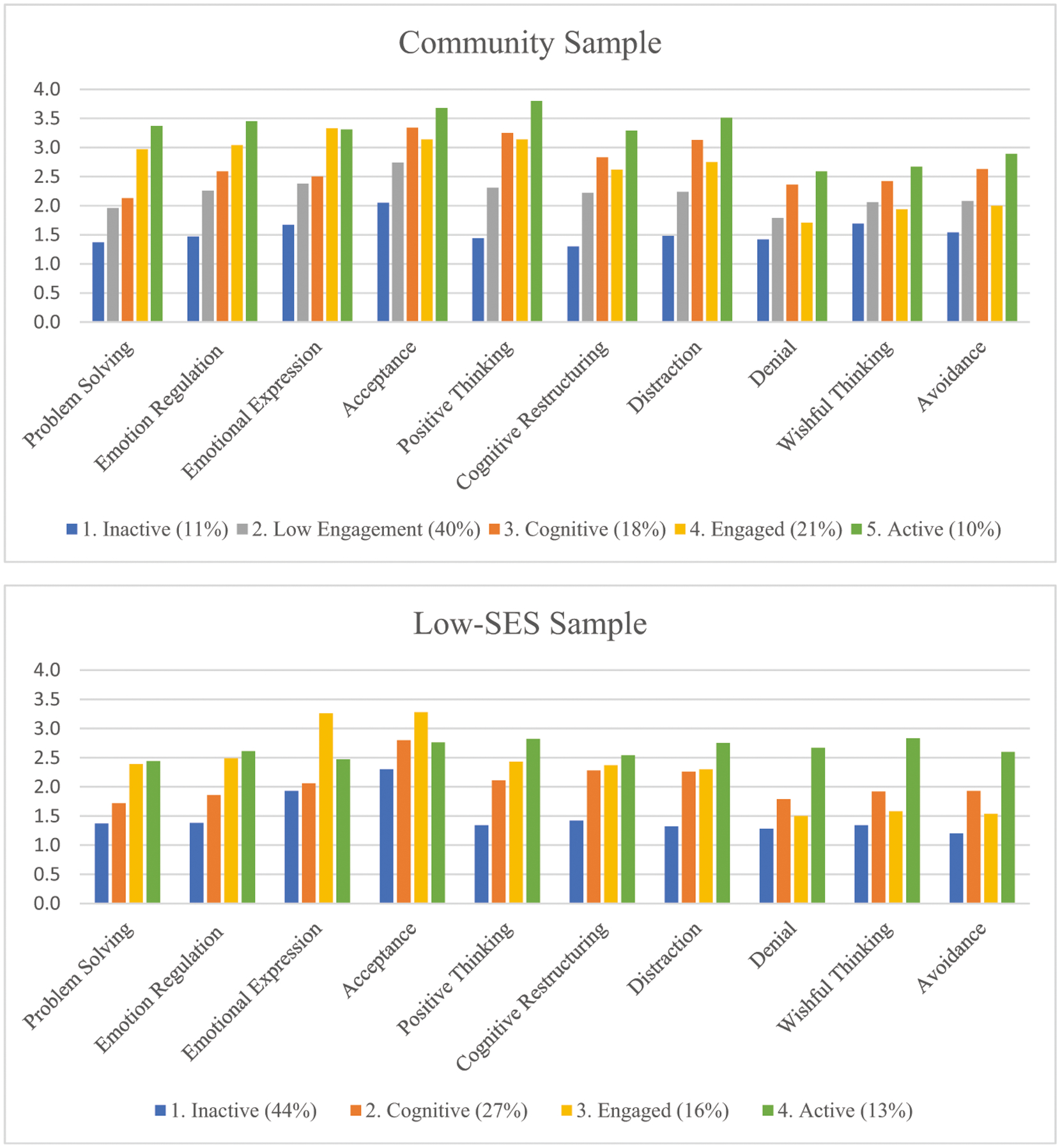
Profile Plots for Both Samples

A summary of the LPA parameter estimates for the profiles identified in the community sample, including item-response means and latent profile membership probabilities, is shown in Table 2. Item-response means suggested the following labels for the five profiles of coping: Inactive Copers, Low Engagement Copers, Cognitive Copers, Engaged Copers, and Active Copers. Inactive Copers (11% prevalence) were characterized by below average reported use of all ten coping strategies. Low Engagement Copers (40%) were characterized by below average low use of most engagement strategies and average use of disengagement strategies. Cognitive Copers (18%) were characterized by average use of active engagement strategies and above average use of cognitive engagement and disengagement strategies. Engaged Copers (21%) were characterized by above average use of active engagement and cognitive engagement strategies and below average use of disengagement strategies. Finally, Active Copers (10%) were characterized by above average use of all ten coping strategies. These profiles are depicted in Fig. 2.

### Low-SES Sample

Model fit information and selection criteria for the latent profile models of coping suggested four profiles appeared superior to models with fewer or greater profiles. This supports the hypothesis that at least three coping profiles would be identified. The 4-profile model showed greater profile separation compared to the 3-profile model, and the additional profile in the larger model improved interpretation. In contrast, when moving from the 4-profile model to the 5-profile model, one very small profile that was not clearly or conceptually distinct from the other four profiles was identified (eight participants, 3% of the sample). Thus, the 4-profile model was selected as optimal for interpretation and further analysis.

A summary of the LPA parameter estimates for the profiles identified in the low-SES sample, including item-response means and latent profile membership probabilities, is shown in Table 3. Item-response means suggested the following labels for the four latent profiles of coping among low-SES adolescents: Inactive Copers, Cognitive Copers, Engaged Copers, and Active Copers. Inactive Copers (44%) were characterized by below average reported use of all ten coping strategies. Cognitive Copers (27%) were characterized by above average levels of cognitive restructuring, distraction, and avoidance, and average levels of all other strategies. Engaged Copers (16%) were characterized by above average reported use of active engagement strategies and cognitive engagement and average use of disengagement strategies. Finally, Active Copers (13%) were characterized by above average use of all ten coping strategies. These profiles are depicted in Fig. 2.

### Descriptive Differences Between Profiles

Intraprofile means and pairwise comparisons between profiles in the community sample for age, gender, and internalizing psychopathology symptoms are presented in Table 4. It was hypothesized that profiles would differ by participant age, but age was not significantly associated with profile membership overall (*χ* ^2^ = 8.05, *p* = 0.090). Intraprofile pairwise comparisons suggested that the community sample Cognitive Copers may be younger than Active Copers and Low Engagement Copers but not different in age from Inactive or Engaged Copers. Consistent with hypotheses, gender was globally and significantly associated with profile membership (*χ*^2^ = 28.11, *p* < 0.001). The community sample Inactive Copers were more likely to be male than Low Engagement, Cognitive, Engaged, and Active Copers, and the Engaged Copers profile had the highest proportion of girls. Consistent with hypotheses, psychopathology was also globally and significantly associated with profile membership for the community sample (CDI: *χ*^2^ = 30.89, *p* < 0.001, MASC: *χ*^2^ = 14.17, *p* = 0.007). The community sample Engaged Copers reported lower depressive symptoms as compared to all other profiles, and the Inactive Copers reported lower symptoms of anxiety as compared to all other profiles. Active Copers reported the highest levels of internalizing symptoms, though this difference was only significantly higher for depression as compared to the Engaged Copers and for anxiety as compared to the Inactive Copers.

Intraprofile means and pairwise comparisons between profiles in the low-SES sample for age, gender, and psychopathology are also presented in Table 4. It was hypothesized that profiles would differ by participant age, but again age was not significantly associated with profile membership overall (*χ*^2^ = 6.62, *p* = 0.085). Intraprofile pairwise comparisons suggested that the low-SES Active Copers may be younger than Inactive and Engaged Copers but not different in age from Cognitive Copers. Consistent with hypotheses, gender was globally and significantly associated with profile membership (*χ*^2^ = 13.92, *p* = 0.003). The low-SES Inactive Copers were more likely to be male than Engaged, Active, and Cognitive Copers, and the Engaged Copers profile had the highest proportion of girls. Consistent with hypotheses, psychopathology was also globally and significantly associated with profile membership for the low-SES sample (*χ*^2^ = 28.42, *p* < 0.001). The low-SES Active Copers reported the highest level of anxious/depressed symptoms. This profile’s level of anxious/depressed symptoms was higher than all other profiles. Cognitive Copers additionally reported higher anxious/depressed symptoms as compared to Inactive and Engaged Copers. Engaged Copers reported the lowest symptom levels, though this difference was only significantly lower than the Active Copers profile.

## Discussion

Coping is a multifaceted process involving numerous co-occurring strategies (Skinner et al. 2003), and individual differences in patterns of coping are meaningfully associated with psychosocial outcomes during adolescence (e.g., Herres 2015). Adolescent coping repertoires develop in context, and patterns or profiles of coping that are adaptive in some contexts may be maladaptive in others (Wadsworth 2015). Prior studies have applied person-centered methods to study adolescent coping, but little is known about similarities and differences in adolescent coping profiles across contexts. The present study compared coping profiles across two distinct samples of adolescents at varying levels of contextual risk using LPA: a nationally representative community sample and a rural, low-SES sample. Further, pairwise profile comparisons were conducted to investigate descriptive differences between coping profiles on measures of age, gender, and internalizing psychopathology. Findings highlight that, although adolescent coping patterns are generally comparable in these two distinct samples, there are meaningful differences in profile prevalences and in profile associations with symptoms of internalizing psychopathology across contexts. Multidimensional coping profiles may be more informative than broad coping factors if the goal is to increase intervention effectiveness. Applying findings from person-centered research like this study may increase program effectiveness by helping to identify individuals who are most likely to benefit (Kaluza 2000) and by uncovering which intervention targets are most needed.

LPA results suggested that five coping profiles were evident in the community sample: Inactive Copers (11%), Low Engagement Copers (40%), Cognitive Copers (18%), Engaged Copers (21%), and Active Copers (10%). Four of these coping profiles were evident for low-SES adolescents: Inactive Copers (44%), Cognitive Copers (27%), Engaged Copers (16%), and Active Copers (13%). The Low Engagement Copers profile was unique to the community sample, whereas all other profiles were identified in both samples. These coping profiles are similar to profiles that have been identified in previous studies. Current findings extend previous understanding of individual differences in adolescent coping across contexts by elucidating within-person patterns of coping in two distinct samples of adolescents with varying levels of poverty-related stress and by allowing for comparison of similarities and differences in coping patterns between these two groups.

Research examining within-person patterns of coping is in its infancy; there are only a few studies available to inform considerations of reliability and replicability of coping profiles. Previous studies of within-person differences in adolescent coping repertoires have typically uncovered 3–4 profiles distinguished by different combinations of approach and avoidance coping strategies. The studies to date that have applied person-centered methods to elucidate within-person coping patterns have typically focused on specific populations of adolescents, such as adolescents of color (Aldridge and Roesch 2008) and adolescents from military families (Okafor et al. 2016). The current study investigates coping in two distinct samples: a narrower sample of rural, low-SES adolescents and a broader and more racially and socioeconomically representative community sample. Studies that have included broader samples may have been limited by samples sizes too small to identify more profiles. Additionally, no prior person-centered study has assessed adolescent coping with the RSQ, which measures coping across ten behavioral domains that assess engagement as well as disengagement strategies.

The study that most closely resembles the present community sample did have a sample large enough to identify multiple profiles (*N* = 982) but focused on older adolescents (*M*_age_ = 16.04; Herres 2015). The study that most closely resembles the present low-SES sample was comparable in terms of sample size, age, gender, and SES, but was characterized by much higher racial diversity than the present low-SES sample (Aldridge and Roesch 2008). Youth of color face unique stressors including racial discrimination and acculturation stress that White youth do not experience (Grant et al. 2000). Adolescents of color also may rely more on avoidance coping strategies than White adolescents, in part because these strategies may be more adaptive for marginalized youth facing high levels of uncontrollable or systemic stress (Gonzales et al. 2001). This may explain the small but distinct Avoidant Copers profile in Aldridge and Roesch’s (2008) study that was not identified in the present study. McDermott et al. 2019 identified a similar profile in a study investigating how Latinx adolescents cope with ethnic-racial discrimination specifically. This profile, labeled “Passive and Moderately Proud” reported frequently ignoring situations involving discrimination and feeling moderately proud of themselves. Consistent with Aldridge and Roesch’s (2008) interpretation, these findings also suggest that disengaging from uncontrollable stressors may be adaptive for youth of color in particular, at least in the short term. Notably, though this profile of adolescents reported a moderate sense of pride, they also endorsed lower self-esteem and academic motivation than another profile of adolescents, labeled “Proactive” and characterized by higher levels of engagement strategies including talking through discrimination, working hard to prove others wrong, and a higher sense of pride than the “Passive and Moderately Proud” profile. Further research that elucidates coping patterns across racially and ethnically diverse samples of youth facing adversity is needed, as much of the available evidence suggests that there are racial and ethnic differences in the effectiveness of coping in stressful contexts as well as variability in how coping and psychosocial outcomes are associated in different adolescent populations facing similar environmental stressors (Costello et al. 2001).

Despite differences in sampling and methodology, the profiles identified in the present study are similar in many ways to those that have been uncovered previously. All these studies have identified a group of adolescents comparable to the Inactive Copers that reported low use of all strategies. Most previous studies have also identified a group of adolescents that reported high use of all strategies, comparable to the Active Copers. Previous studies have also consistently identified a profile of adolescents characterized by active, approach, or engagement strategies comparable to the Engaged Copers (Aldridge and Roesch 2008; Herres 2015; Okafor et al. 2016; Seiffge-Krenke and Klessinger 2000). The profiles identified in the sample of low-SES adolescents were also generally similar to profiles of stress responses identified in a sample of racially diverse, low-SES parents using the RSQ (Perzow et al. 2018).

Two of the profiles identified in this study are notably different than groups that have been discussed in the literature to date. The Low Engagement Copers (identified only in the community sample), who broadly reported low levels of engagement strategies and average levels of disengagement, and the Cognitive Copers, who endorsed high reliance on cognitive engagement and disengagement strategies, are previously unidentified in the person-centered coping literature. These subgroups of adolescents may be larger and more stable in the present, generally representative, study samples than in narrower samples. The Low Engagement Copers profile may also only occur in lower-stress contexts that present fewer coping demands. These subgroups may also have been previously encompassed by other subgroups because of differences in measurement of coping.

### Comparing Coping Profiles Across Contexts

Coping profiles identified in the community and low-SES samples of adolescents are more similar than they are different. Each sample evidenced a clearly defined profile of adolescents that reported very low use of all coping strategies (Inactive Copers) and a contrasting profile that reported very high reliance on all coping strategies (Active Copers). Both samples also evidenced a profile of adolescents who employed high active and cognitive engagement strategies and relied less on disengagement strategies (Engaged Copers). Finally, a Cognitive Copers profile that was characterized by high cognitive engagement and disengagement strategies was evident in both samples. In summary, the patterns of responses that distinguished the Inactive, Engaged, Cognitive, and Active Copers profiles were largely comparable in these two samples of adolescents.

Some differences between samples should be expected because these samples represent distinct populations with different levels of stress exposure and consequently different contextual demands for coping. Expected differences include indicator means and profile prevalences. Other differences, such as the identification of different profiles or different patterns between profiles across studies, require closer consideration. The primary difference between profiles in these two samples is the absence of a Low Engagement Copers profile in the low-SES sample, suggesting that there are two distinct patterns of low-level coping in the community sample and just one low-level coping pattern in the low-SES sample. Close investigation of the Inactive and Low Engagement profiles in the community sample confirmed that they are distinct; the Low Engagement Copers report below average use of engagement strategies and average levels of disengagement strategies whereas the Inactive Copers report very low use of all coping strategies including disengagement strategies. This difference between samples may be best understood by considering differences in stress exposure for the two samples, with the low-SES sample experiencing higher stress due to poverty and the community sample being exposed to lower contextual stress and therefore likely to include a larger proportion of adolescents who require less coping overall and may be able to cope with stressors with more disengagement than adolescents living in higher stress contexts; this pattern is evident in the Low Engagement profile.

A greater proportion of adolescents in lower-stress contexts report low-level coping, perhaps because they face less stress and thus lower demand to cope. Temporal and geo-graphical differences between these two samples also may contribute to divergence in profiles identified in the present study. Data collection for the Adolescents Coping with Poverty project (the low-SES sample) began twelve years before data collection for the GEM Study (community sample), and the low-SES sample was recruited from a rural region in the northeastern US whereas the community sample was recruited in suburban and urban areas in the western and eastern US. Notably, means on all ten of the RSQ subscales were lower for the low-SES sample than the community sample. This is somewhat consistent with previous research demonstrating that PRS is negatively associated with engagement coping but is inconsistent with findings that PRS is positively associated with disengagement coping (Wadsworth and Compas 2002). It is also notable that the Inactive Copers profile in the low-SES sample represented a large subset of the sample (44%), whereas the Inactive Copers profile in the community sample was much smaller (11%). The extent to which adolescents engage in coping is likely due to the level and type of stress that they are exposed to, and these profiles may be distinguished by different environmental coping demands. A larger proportion of the low-SES sample may reflect a blunting effect if they are facing stressors so lofty or uncontrollable that no coping efforts seem to help. An inactive coping pattern may be more adaptive in rural, low-income environments characterized by greater exposure to uncontrollable stressors such as higher rates of poverty and unemployment (United States Department of Agriculture 2019), greater geographic isolation (Hudson and Doogan 2019), and vast disparities in mental and physical health (Meit et al. 2014). Future research is needed to investigate the associations between stress exposure and profile membership.

### Descriptive Differences between Coping Profiles

Profiles differed in meaningful and informative ways on measures of gender but not age. Inactive Copers were more likely than members of other coping profiles to be boys; a result that is consistent with previous study findings that adolescent (Herres 2015) and adult (Perzow et al. 2018) men endorse less coping behavior than women do. Alternatively, previous research has also found gender differences in adolescent perceived stress, with girls endorsing higher stress than boys (Hampel and Petermann 2005). Men may perceive less stress in their environments and as a result may engage less in coping. Another possible explanation for gender differences may be that gendered socialization of youth coping encourages emotional expression and support seeking in girls more than boys, while boys may be encouraged to suppress or disengage from their feelings or finding outlets to help themselves feel better. The current study provides support for this possibility, as profiles endorsing lower coping efforts had a higher proportion of boys and profiles endorsing greater coping efforts had a higher proportion of girls. These differences by gender and perceived stress should be examined further in future research.

There were no overall age differences between profiles in this study. Previous studies have similarly found small or nonsignificant age effects in adolescent coping profiles (Aldridge and Roesch 2008; Herres 2015; Okafor et al. 2016). Still, possible age effects should not be dismissed in future studies. Most previous studies have been cross-sectional and longitudinal data will be helpful for capturing the effects of maturation on coping skill acquisition. More prospective, longitudinal studies will help to elucidate age-related differences between coping profiles.

Profiles differed meaningfully on measures of psychopathology. Engaged Copers reported the lowest levels of anxious/depressed symptoms in the low-SES sample. The Engaged Copers also reported lower symptoms of depression and anxiety in the community sample; only the Inactive Copers reported lower anxiety symptoms, and the Engaged Copers reported the lowest level of depressive symptoms in the community sample. Thus, the pattern of high engagement and low disengagement coping strategies observed in the Engaged Copers profile appears to be protective against psychopathology across contexts. This is consistent with previous research indicating that engagement coping can be protective while disengagement can be maladaptive (Compas et al. 2017). In contrast, Active Copers may be at higher risk for psychopathology than might be expected. Active Copers reported the highest levels of internalizing psychopathology across all measures in these two distinct samples. High levels of engagement strategies may not be protective when used in combination with high levels of denial and wishful thinking. It is also possible that the Active Copers profile reflects high levels of indiscriminate coping activity necessitated by higher levels of stress. Finally, the Cognitive Coping profile reported higher anxious/depressed symptoms as compared to the Inactive and Engaged Copers in the low-SES sample. The Cognitive Copers reported higher symptoms of anxiety as compared to the Inactive Copers in the community sample but did not differ from these other profiles in their reported level of depressive symptoms. The combination of predominantly cognitive coping strategies including avoidance (a disengagement strategy) seems to be more adaptive in lower stress environments but may become maladaptive in the face of greater chronic, uncontrollable stress such as PRS faced by the adolescents in the low-SES sample.

### Limitations and Future Directions

Results of this study are presented with recognition of limitations. First, measurement of all indicators relied on adolescent self-report. Self-report measures are valid and appropriate in many circumstances, particularly for assessing and measuring internal cognitive processes such as beliefs, attitudes, moods, and emotions (Haeffel and Howard 2010). Still, future studies may consider incorporating reports from multiple informants to limit bias that can arise from shared method variance. Parent-report of adolescent coping, for example, may measure more observable coping strategies or strategies that parents encourage their children to use (as opposed to strategies adolescents report using). Further, concordance between parent-report and self-report of coping may be a marker of parent-child relationship or family socialization of coping. Future research that includes both parent and adolescent self-report of coping using person-centered methods may contribute substantially to research into parenting, parent-child relationships, and family functioning.

Further, studies that more thoroughly examine the role of stress in the development and adaptiveness of coping profiles are needed. Coping is specifically an effortful response to environmental stress, and the raw subscale scores included in present analyses do not account for total coping efforts or for level of stress exposure. Future analyses that consider ways to use ratio scores (Connor-Smith et al. 2000) are needed. Research that examines the unique effects of perceived stress and stress exposure is also needed. Additionally, not all youth living in low-SES environments perceive high levels of stress or face equal exposure to environmental stress. The current low-SES sample represents a specific population of predominantly White adolescents residing in an impoverished, rural part of the Northeastern United States. Research investigating patterns of coping in more diverse, representative samples of low-SES adolescents is needed.

Finally, the current study is cross-sectional and cannot elucidate how coping profiles develop across contexts. This developmental process likely varies between low-and high-stress environments as well as between distinct high-stress environments. Recent compelling research by Rabinowitz et al. (2020) suggests that there are likely differences in the development of coping repertoires low-income predominantly African American young adults (*M*_age_ = 18.76 years) depending on the level of neighborhood violence and disadvantage. Specifically, young adults residing in neighborhoods characterized by low cohesion and high disorder, violence, and disadvantage reported higher levels of positive cognitive restructuring and problem-focused coping as compared to young adults in neighborhoods characterized by less cumulative disadvantage. Longitudinal research elucidating the development of coping profiles across contexts in more diverse samples is clearly needed to better understand how, when, and for whom coping is most adaptive or maladaptive.

Integration of person-centered methods in applied behavioral research is gaining traction. Studies across disciplines with varied samples are accumulating that encourage the adoption of these methods for designing and evaluating research, for example by providing clinical utility for selecting the most appropriate level of intensity of services for youths based on their clinical subgroup (Bonadio and Tompsett 2018), uncovering subgroups of young readers who are most likely to improve following a web-based tutoring program (Ji et al. 2018), and identifying subgroups of Coronary Artery Disease patients with varying levels of engagement in rehabilitation efforts (van Montfort et al. 2018). One size does not fit all, though interventions often assume so. Evidence-based programs are superior to treatment-as-usual, though these interventions often have small effect sizes (Weisz et al. 2006). Intervention programs might have larger effects if they accounted for evidence from person-centered studies. Resulting programs may better accommodate adolescents’ different strengths and weaknesses, pre-existing skills, and areas for improvement.

## Conclusion

Adolescent development can be promoted through stress-related growth (Mansfield and Diamond 2015) or hindered by exposure to chronic and uncontrollable stress (Wadsworth et al. 2016). Coping is a malleable mechanism that can mitigate the psychological consequences of excessive stress (Compas et al. 2017), but to date research that elucidates differences in adolescent coping repertoires across individuals and contexts is sparse. Application of person-centered methods such as LPA, which models interactions among variables within individuals, to compare coping patterns across contexts is a promising avenue for elucidating individual differences in coping and for linking these patterns or profiles with psychosocial outcomes. The present investigation expands current understanding of adolescent coping by applying LPA to identify and describe coping profiles in two distinct samples of adolescents: a community sample and a rural, low-SES sample. Coping patterns were generally similar in these disparate samples of adolescents, with Inactive, Engaged, Cognitive, and Active Copers profiles identified in both. A Low Engagement Copers profile was also identified in the community sample, suggesting that while adolescents employ similar coping strategies across contexts, fewer low-SES adolescents engage in low-level coping. Coping profiles were associated with gender and internalizing psychopathology. Inactive Copers were more likely to be male in both samples, suggesting that gendered socialization of coping processes may be similar across contexts. Engaged Copers in both samples reported the lowest symptom levels, while Active Copers reported elevated symptoms. Adolescents who engage in high levels of engagement as well as disengagement strategies may be at higher risk for internalizing psychopathology. Cognitive Copers reported higher anxious/depressed symptoms in the low-SES sample only, suggesting that adolescents who engage in higher levels of cognitive restructuring, distraction, and avoidance may be at increased risk for psychopathology in the context of greater PRS, while this pattern of coping may be more adaptive in less stressful contexts. One of the most meaningful contributions of the present study and others that investigate individual differences in malleable mechanisms such as coping is that these studies uncover subgroups of individuals with similar patterns of behaviors that can be targeted by tailored interventions. Adolescents in different contexts likely will benefit most from interventions that consider the unique demands of distinct environments and the behavioral presentations that are most associated with resilience to context-specific stress. Future studies that apply these methods to developing and evaluating interventions are needed. The result may be more effective, more cost-efficient, and better received behavioral interventions.

## Figures and Tables

**Table 1:** Fit indices for latent profile models of coping

No. of Profiles	No. of Free Parameters	Log-Likelihood	AIC	BIC	a-BIC	a-BIC Difference	Entropy	VLMR	BLRT
*Community Sample (N = 374)*									
1	20	−4120	8280	8358	8295	–	–	–	–
2	31	−3701	7464	7585	7487	808	0.83	<0.0001	<0.0001
3	42	−3553	7189	7354	7221	266	0.85	0.058	<0.0001
4	53	−3476	7058	7267	7098	122	0.84	0.025	<0.0001
**5**	**64**	**−3420**	**6969**	**7220**	**7017**	**82**	**0.84**	**0.051**	**<0.0001**
6	75	−3378	6907	7201	6963	53	0.82	0.461	<0.0001
7	86	−3349	6870	7207	6934	29	0.83	0.190	<0.0001
8	97	−3324	6842	7222	6914	20	0.82	0.759	<0.0001
*Low-SES Sample (N = 304)*									
1	20	−3388	6817	6891	6828	–	–	–	–
2	31	−2990	6041	6156	6058	769	0.89	<0.0001	<0.0001
3	42	−2867	5819	5975	5842	217	0.89	0.001	<0.0001
**4**	**53**	**−2816**	**5738**	**5935**	**5767**	**75**	**0.87**	**0.620**	**<0.0001**
5	64	−2779	5687	5924	5721	45	0.89	0.174	<0.0001
6	75	−2747	5645	5923	5685	36	0.89	0.205	<0.0001
7	86	−2716	5604	5923	5651	35	0.89	0.136	<0.0001
8	97	−2693^a^	5579	5940	5632	18	0.88	0.789	0.002

Bold texts indicates selected model

a The best log-likelihood value was not replicated

**Table 2:** Parameter Estimates for the 5-Profile LPA of Coping for the Community Sample

		(1) Inactive	(2) Low Engagement	(3) Cognitive	(4) Engaged	(5) Active
		*Latent Profile Membership Probabilities*				
Item	Sample Mean (SD)	*0.11 (n = 42)*	*0.40 (n = 149)*	*0.18 (n = 66)*	*0.21 (n = 78)*	*0.10 (n = 39)*
		*Item-Response Means*				
Problem Solving	2.29 (0.83)	1.37^−^	1.96^−^	2.13	2.97^+^	3.37^+^
Emotion Regulation	2.52 (0.73)	1.47^−^	2.26^−^	2.59	3.04^+^	3.45^+^
Emotional Expression	2.62 (0.78)	1.67^−^	2.38^−^	2.50	3.33^+^	3.31^+^
Acceptance	2.95 (0.70)	2.05^−^	2.74^−^	3.34^+^	3.14	3.68^+^
Positive Thinking	2.71 (0.83)	1.44^−^	2.31^−^	3.25^+^	3.14^+^	3.80^+^
Cognitive Restructuring	2.42 (0.79)	1.30^−^	2.22^−^	2.83^+^	2.62^+^	3.29^+^
Distraction	2.55 (0.75)	1.48^−^	2.24^−^	3.13^+^	2.75	3.51^+^
Denial	1.92 (0.63)	1.42^−^	1.79^−^	2.36^+^	1.71^−^	2.59^+^
Wishful Thinking	2.12 (0.73)	1.69^−^	2.06	2.42^+^	1.94^−^	2.67^+^
Avoidance	2.18 (0.65)	1.54^−^	2.08	2.63^+^	2.00^−^	2.89^+^

Values significantly different than sample mean indicated by ^+^ (significantly above sample mean) and ^−^ (significantly below sample mean) *p* < 0.05

**Table 3:** Parameter Estimates for the 4-Profile LPA of Coping for the low-SES Sample

		(1) Inactive	(2) Cognitive	(3) Engaged	(4) Active
		*Latent Pro*fi*le Membership Probabilities*			
Item	Sample Mean (SD)	*0.44 (n = 134)*	*0.27 (n = 82)*	*0.16 (n = 49)*	*0.13 (n = 40)*
		*Item-Response Means*			
Problem Solving	1.77 (0.76)	1.37^−^	1.72	2.39^+^	2.44^+^
Emotion Regulation	1.85 (0.74)	1.38^−^	1.86	2.49^+^	2.61^+^
Emotional Expression	2.25 (0.82)	1.93^−^	2.06	3.26^+^	2.47
Acceptance	2.65 (0.86)	2.30^−^	2.80	3.28^+^	2.76
Positive Thinking	1.92 (0.76)	1.34^−^	2.11	2.43	2.82^+^
Cognitive Restructuring	1.95 (0.75)	1.42^−^	2.28^+^	2.37	2.54^+^
Distraction	1.92 (0.77)	1.32^−^	2.26^+^	2.30^+^	2.75^+^
Denial	1.63 (0.63)	1.28^−^	1.79	1.50	2.67^+^
Wishful Thinking	1.73 (0.75)	1.34^−^	1.92	1.58	2.83^+^
Avoidance	1.63 (0.59)	1.20^−^	1.93^+^	1.54	2.60^+^

Values significantly different than sample mean indicated by ^+^ (significantly above sample mean) and ^−^ (significantly below sample mean) *p* < 0.05

**Table 4:** Intraprofile Means for Comparison Measures

*Community Sample*						
		(1) Inactive	(2) Low Engagement	(3) Cognitive	(4) Engaged	(5) Active
	Sample Mean (SD)	*0.11 (n = 42)*	*0.40 (n = 149)*	*0.18 (n = 66)*	*0.21 (n = 78)*	*0.10 (n = 39)*
Age*	13.14 (1.62)	12.93	13.35^c^	12.76^b,e^	13.23	13.65^c^
Gender**	0.57 (0.50)	0.31^b,c,d,e^	0.54^a,d^	0.63^a^	0.78^a,b^	0.67^a^
CDI Total Score	7.29 (5.90)	8.07^d^	7.98^d^	6.95^d^	4.29^a,b,c,e^	9.14^d^
MASC Total Score	40.55 (14.80)	32.78^b,c,d,e^	40.36^a^	42.32^a^	39.00^a^	45.26^a^

*Low-SES Sample*					
		(1) Inactive	(2) Cognitive	(3) Engaged	(4) Active
	Sample Mean (SD)	*0.44 (n = 134)*	*0.27 (n = 82)*	*0.16 (n = 49)*	*0.13 (n = 40)*
Age*	14.56 (1.56)	14.75^e^	14.51	14.84^e^	13.95^a,d^
Gender**	0.55 (0.50)	0.43^c,d,e^	0.59^a^	0.72^a^	0.64^a^
YSR Anxious/Depressed	54.06 (6.79)	52.23^c,e^	54.30^a,e^	52.21^e^	61.71^a,c,d^

* Overall test non-significant.

** Proportion female. Significantly different from:

a Inactive,

b Low Engagement,

c Cognitive,

d Engaged,

e Active *p* < 0.05
